# Increased CSF aquaporin-4, and interleukin-6 levels in dogs with idiopathic communicating internal hydrocephalus and a decrease after ventriculo-peritoneal shunting

**DOI:** 10.1186/s12987-016-0034-1

**Published:** 2016-06-29

**Authors:** Martin J. Schmidt, Christoph Rummel, Jessica Hauer, Malgorzata Kolecka, Nele Ondreka, Vanessa McClure, Joachim Roth

**Affiliations:** Department of Veterinary Clinical Sciences, Small Animal Clinic, Justus-Liebig-University, Frankfurter Strasse 108, 35392 Giessen, Germany; Institute for Veterinary Physiology and Biochemistry, Justus-Liebig-University, Frankfurter Strasse 100, 35392 Giessen, Germany; Department of Companion Animal Clinical Studies, Faculty of Veterinary Science, University of Pretoria, Private Bag X04, Onderstepoort, Pretoria, 0110 Republic of South Africa

**Keywords:** Aquaporin, Communicating hydrocephalus, Dogs, Interleukin-6

## Abstract

**Background:**

Studies in animal models, in which internal hydrocephalus has been induced by obstructing the cerebrospinal fluid pathways, have documented an up-regulation of the concentrations of aquaporin-4 (AQP4) in the brain. In this study, the concentrations of aquaporin-1 (AQP1), AQP1, AQP4 and interleukin-6 (IL-6) were determined in the CSF of dogs with idiopathic communicating hydrocephalus before and after the reduction of intraventricular volume following ventriculo-peritoneal shunt (VP-shunt) treatment.

**Results:**

The concentrations of AQP4 and IL-6 were increased in the cerebrospinal fluid of dogs with hydrocephalus compared to controls. Both parameters significantly decreased after surgical treatment, accompanied by decrease of ventricular size and the clinical recovery of the dogs. AQP1 was not detectable in CSF.

**Conclusions:**

Brain AQP4 up-regulation might be a compensatory response in dogs with hydrocephalus. Future determination of AQP4 at the mRNA and protein level in brain tissue is warranted to substantiate this hypothesis.

## Background

Definitive treatment options for internal hydrocephalus in dogs and humans rely mostly on surgical implantation of CSF-draining shunt systems [1–4]. Medical treatment methods aiming at reducing CSF production have been reported to provide temporary relief of clinical signs, but are mostly ineffective [2, 5–7]. Therefore, improved medical treatment options, especially for immature hydrocephalic patients, are desirable. Aquaporins (AQPs) are a family of water channel proteins, which are found in cell membranes in a number of tissues, including the central nervous system (CNS) [8, 9]. In the brain, the predominant water channel is AQP4, which provides the molecular basis for bidirectional water transport across the cell membranes of the blood–brain- and blood-CSF boundaries. The driving force behind water movement can be both osmotic and hydraulic in nature, the latter allowing the bulk flow of water across cell membranes [9–11]. AQP4 is mainly located in the astrocyte foot processes that surround capillaries in the CNS. At this boundary, brain AQP4 regulates water removal from the pericapillary space into brain vessels suggesting it has a crucial role in fluid volume homeostasis [8–11].

Under certain pathological conditions changes in AQP4 expression have been found [12–14]. Studies in animal models, in which internal hydrocephalus has been induced by injection of kaolin into the subarachnoid space, documented an up-regulation of AQP4 in the periventricular white matter [15, 16] and cerebral cortex [17]. Increased levels of soluble AQP4 were also found in the CSF of children with naturally occurring internal hydrocephalus [18]. It has been suggested that increased integration of AQP4 into the astrocyte membranes might have a compensatory effect in countering excess CSF [15, 19, 20] by increased astroglial clearance of excess brain water by transcellular routes and/or through the glia limitans [16].

A second water channel in the CNS, namely AQP1, is reported to be limited to choroid plexus epithelia within the CNS. Experimental depletion of the AQP1 gene in mice leads to a decrease in CSF production by the choroid plexus, demonstrating a role for AQP1 in CSF production [21]. The proposed adaptive and protective roles of AQP1 and-4 as regulators of CSF production and absorption in the pathophysiology of hydrocephalus, establishes these AQPs as interesting candidates for possible treatment options. Interleukin 6 (IL-6) is a pro-inflammatory cytokine. Increased levels of IL-6 have been associated with periventricular white matter damage [22]. Neurons, astrocytes, microglia and endothelial cells are the main sources of IL-6 in the CNS [23]. Internal hydrocephalus is primarily a white matter disease and IL-6 levels may be used as a surrogate marker for white matter injury. Thus, a correlation between IL-6 and AQP4 levels could be useful to indicate the extent of white matter damage.

The aim of the present study was to determine the concentration of AQP1and AQP4 in the CSF of dogs with idiopathic communicating hydrocephalus, and possible changes in these levels after reduction of intra-ventricular pressure following ventriculo-peritoneal shunting. Given the previously-reported relationship between IL-6 and white matter damage in neonatal children and the presence of white matter damage in internal hydrocephalus, we also investigated the relationship between IL-6 and the aquaporins. We hypothesised that increased ventricular volume is positively correlated to CSF levels of AQP4 and IL-6, and negatively correlated to AQP1 in dogs with idiopathic communicating hydrocephalus.

## Methods

### Animals

Fourteen dogs with internal hydrocephalus, whose owners decided on surgical implantation of a ventriculo-peritoneal shunt (VP-shunt) system for permanent CSF drainage were prospectively selected between 2010 and 2014. The dogs had to meet the following criteria to be included in the study. (1) The hydrocephalus had to be diagnosed as idiopathic communicating, based on magnetic resonance imaging (MRI) of the brain without signs of parenchymal contrast enhancement or visual obstruction of CSF pathways. (2) Clinical re-evaluation, with CSF samples taken and immediately stored at −80 °C and both pre- and post-operative MRIs performed in our clinic to allow direct comparisons. (3) Medical pre-treatment using drugs to reduce CSF production (glucocorticoids, omeprazole, acetazolamide, furosemide) excluded dogs from the study. (4) In the follow up MRI, a decrease in ventricular volume had to be demonstrated as an indication for effective shunt treatment.

CSF collection and MRI examination was also performed on a control group of 10 dogs that were donated to the clinic after euthanasia due to non-neurological diseases. CSF was taken within 2 min of death of the animals. Ventriculomegaly was an exclusion criterion for the dogs in this group.

### Clinical evaluation

Collected data included the breed, age, gender, body-weight, type and duration of neurological deficits before and after surgery. Clinical data regarding the presenting signs and clinical improvement of the animals after shunting procedures were determined by a board certified neurologist. A standardised neurologic examination was performed prior to, and every day after surgery until discharge, and again 3 months after surgery.

### Magnetic resonance imaging

Diagnosis of idiopathic communicating hydrocephalus was made by MRI using a 1.0 Tesla scanner (Gyroscan Intera, Phillips, Hamburg, Germany) and a solenoid surface coil (C3). For the MRI examination dogs were premedicated with diazepam (0.5 mg/kg IV) and l-methadone (0.5 mg/kg IV). Anesthesia was induced with propofol (4 mg/kg IV) and maintained after endotracheal intubation with isoflurane in oxygen. Sagittal, dorsal, and transverse T2-weighted, transverse FLAIR sequences, T1-weighted before and after intravenous administration of 0.2 mL/kg gadodiamide (Omniscan^®^) were acquired in all animals pre-operatively. The administration of a contrast agent was of course not feasible in the post-mortem scans in the control dogs but was useful to exclude other neurological diseases in the study group. T2-weighted transverse images of the head were chosen from the whole MR-dataset for image segmentation, using T2-Turbospin echo sequences (TE: 120 ms, TR: 2900 ms). Slice thickness varied from 2–3 mm. The field of view measured 180 × 180 mm in small dogs and 210 × 210 mm in large dogs. The matrix was 288 × 288 in small dogs and 384 × 384 in large dogs leading to an in-plane pixel size between 0.625 × 0.625 and 0.54 × 0.54 mm.

**Fig. 1:**
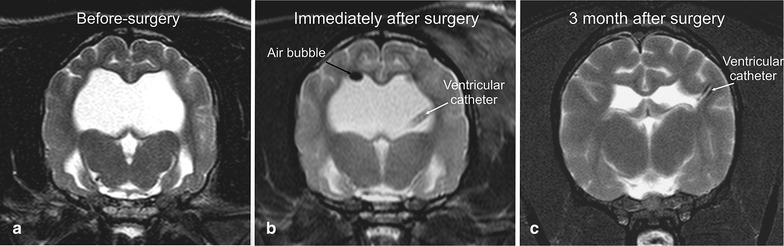
Transversal T2-weighted MR-image of the brain and ventricular system of a bullterrier with internal hydrocephalus at the time of diagnosis (**a**), directly post-operatively (**b**), and 3 months after surgery (**c**) showing the reduction of the ventricular volume and reconstitution of the cerebral parenchyma

Accumulation of CSF in the lateral cerebral ventricles, an absent septum pellucidum, dorsal bulging of the corpus callosum and dilation of the third ventricle all indicated active ventricular distension and were consistent with hydrocephalus [24]. Patency of the mesencephalic aqueduct was assessed in all image planes. A clearly visible hyperintense signal (CSF) within the aqueduct and a non-distended 4th ventricle determined the presence of communicating hydrocephalus [25]. The absence of any other visible lesion and lack of contrast enhancement within the brain parenchyma finally resulted in the diagnosis of idiopathic communicating hydrocephalus. MRI in the control group was without special findings. To assess the ventricular dimensions and restoration of the brain parenchyma, MRI was repeated 3 months after surgery in the study group (Fig. 1).

### Morphometric procedures

**Fig. 2:**
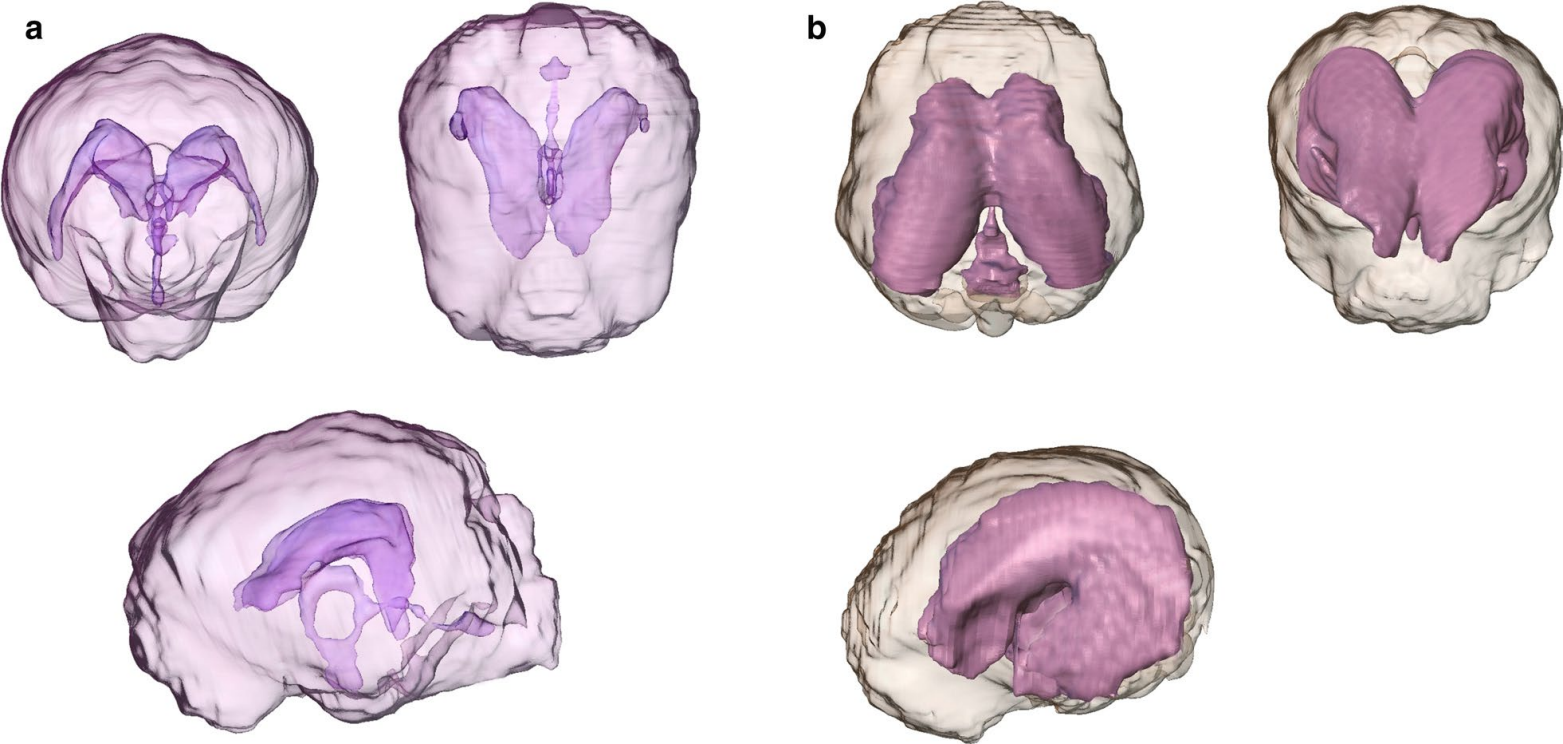
Volumetric determination of the brain and ventricular volume of a control dog (**a**) in contrast to a hydrocephalic dog from the study group (**b**). The brain parenchyma is transparent, allowing the view of the ventricular system

The volume of the cerebral ventricles and the brain tissue was determined based on the T2-weighted images. Image processing for volume rendering was achieved using specialised graphical software as described elsewhere [26] (AMIRA^®^, Mercury Computer Systems, Berlin, Germany), which allowed manual image segmentation of the ventricular system and brain parenchyma on a slice-by-slice basis. The segmented partitions were calculated and graphically presented by the programme (Fig. 2).

### Shunting procedures

**Fig. 3:**
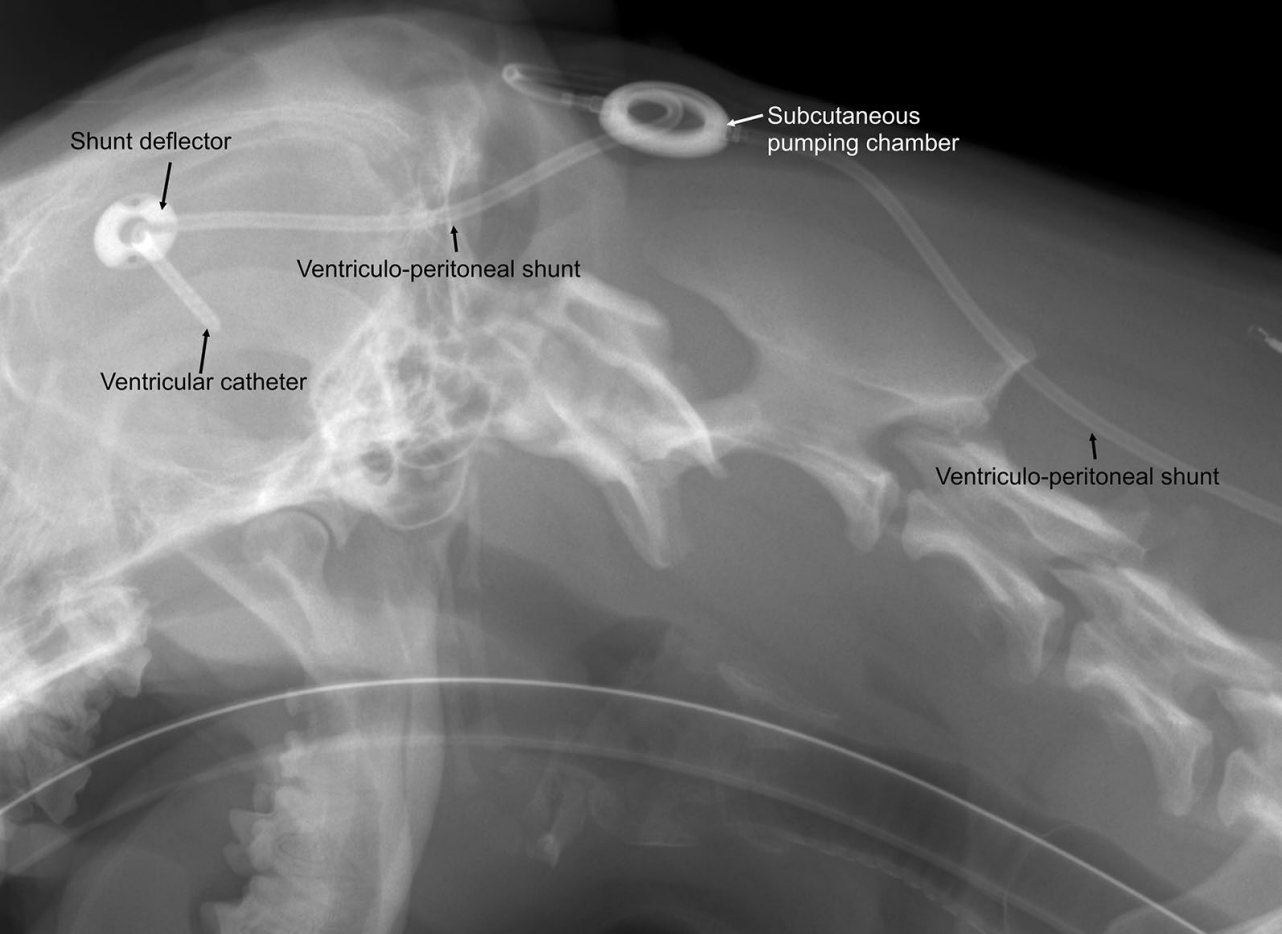
Laterolateral radiograph of the head and neck of a bullterrier after ventriculo-peritoneal shunting showing the proximal components and course of the shunt system

Ventriculo-peritoneal shunting was performed as described elsewhere [3] (Fig. 3 Lateral radiograph of dog with shunt in place). A gravitational ball valve was used in all dogs (paediGAV^®^, Miethke GmbH & Co KG, Potsdam, Germany).

### CSF sampling

On the day of the diagnostic MRI, a routine CSF examination was performed (cytology and biochemical analysis) to rule out inflammatory diseases of the brain. CSF was collected from the cisterna magna (1 mL per 5 kg body weight).

The first CSF specimen for AQP and IL-6 analysis was collected from the pumping chamber during VP-shunt placement. After insertion of the ventricular catheter and connection of the pumping chamber, CSF was allowed to exit the ventricles via the shunt until pulsatile flow was observed in the transparent chamber. Depending on the ventricular volume and the size of the dog, 1–2 mL CSF was collected and stored in plastic tubes (Eppendorf tubes) at −80 °C for further analyses. The ventricular catheter was routinely tested for patency: 3 h after emptying the chamber via transcutaneous puncture, the chamber was checked for refilling by repeat puncture. The second CSF specimen was collected 3 months after shunt insertion from the subcutaneous pumping chamber.

### Ethical approval

Approval by an ethics committee for the CSF collection was not required as all procedures are part of the therapeutic procedure or routine diagnostic workup of clinical patients [German Animal Experiment Act [“Tierschutzgesetz”], paragraph 9.2]. Verbal approval to examine the CSF for research purposes was obtained from the owners.

### CSF AQP1, AQP4, and IL-6 measurements

Frozen CSF specimens were evaluated 4–26 months after sampling. A dog-specific ELISA Kit was used for analyses of AQP1 and AQP4 in CSF, according to the instructions provided by the manufacturer (Canine AQP ELISA kit, assay ID AQP4: E08A0467; AQP1: E08A0863, BlueGene Biotech, Shanghai, China). The kit applied the competitive immunoassay technique using a monoclonal anti-AQP4-antibody and an AQP4 horseradish- peroxidase conjugate. Standards containing 0, 2.5, 5.0, 10, 25 and 50 ng/mL AQP4 were used to create a standard curve, which was used to calculate AQP4 concentrations in the biological samples. According to the manufacturer’s instructions no significant cross-reactivity or interference between AQP4 and its analogues was observed. The coefficient of variation within a given lot and between different lots was stated to be less than 10 %. Samples were determined in duplicate and the detection limit of the specific assay proved to be 0.1 ng/mL.

IL-6 concentrations were determined by a bioassay based on the dose-dependent growth stimulation of IL-6 on the B9 hybridoma cell line [27, 28]. This cell line requires IL-6 for survival and proliferation. The advantages of the B9 assay are its extreme sensitivity and its feature that only bioactive molecules are measured. The assay was performed in sterile, 96-well microtiter plates. In each well, 5000 B9 cells were incubated for 72 h with serial dilutions of biological samples (cerebrospinal fluid) or with different concentrations of a human IL-6 standard (code 89–548, National Institute for Biological Standards and Control, South Mimms, UK). Samples were pre-diluted so that serial dilutions of samples and standard dilution curves were made in parallel. The number of cells in each well was measured by the dimethylthiazol-diphenyl tetrazolium bromide (MTT) colorimetric assay [27]. The detection limit of the assay, after considering the dilution of samples, was set at 3 international units (IU)/mL.

### Statistical analysis

All data was analysed using a statistical software package (Graph Pad Prism 4.0, Graph Pad Software Inc., San Diego, California). AQP and IL-6 concentration before and after surgery were compared with controls. CSF volume before and after surgery may not only be dependent on ventricular distension, but also on the size of the dog (1.2–20 kg, see Table 1, Epidemiological data and clinical signs) as this also influences ventricular volume. Therefore, we considered that the concentrations of AQP4 and IL-6 could also be influenced by CSF volume. To take these morphological differences into account, the concentration of AQPs (ng/mL) and IL-6 (IU/mL) were additionally multiplied by the total ventricular volume (expressed as total AQP/-IL-6 quantity) to calculate the total amounts of AQPs and IL-6 in the ventricles.

**Table 1:** Epidemiological data and results of pre- and postoperative clinical examination of the study group

Number	Breed	Gender, age, bodyweight	Clinical signs	Postoperative clinical signs
1	Boston terrier	Male, 3 months old, 2.5 kg	Obtundation, mild ataxia on all four limbs, circling, aimless barking	None
2	Mini Australian shepherd	Male-neutered, 26 months old, 12.5 kg	Visual deficits, reduced menace, circling	Visual deficits, reduced menace
3	Peruvian hairless dog	Male, 2 months old, 2.8 kg	Ataxia on all four limbs, ventro-lateral strabismus, obtundation	None
4	Austrian hound	Male, 54 months old, 20 kg	Obtundation, circling, head tremor, hypermetria in the front limbs	None
5	Cavalier King Charles spaniel	Male neutered, 60 months old, 9.9 kg	Obtundation	None
6	Pug	Male, 21 months old, 9.2 kg	Obtundation, ataxia on all four limbs	None
7	Papillon	Female, 7 months old 3.4 kg	Obtundation, ataxia, head tilt tremor	None
8	Chihuahua	Male neutered, 3 months old, 1.2 kg	Obtundation, mild ataxia, spasticity in all four limbs, reduced menace response	None
9	Australian shepherd	Male, 4 months old, 13.9 kg	Visual deficits. reduced menace, nystagmus, hypoactive	Visual deficits, reduced menace
10	Jack Russell terrier	Male, 4 months old, 4.7 kg	Obtundation, ataxia on all four limbs	None
11	Bullterrier	Female, 25 months old 12.5 kg	Intermittent obtundation, head pressing	None
12	Maltese mix	Male neutered, 6 months old, 6.1 kg	Circling, obtundation	None

After assessment of the normal distribution of this data using the Shapiro–Wilk test, one-way ANOVA was used to test for global differences between the mean concentration and total AQP4 quantity as well as the ventricular volume before and after surgery. If significant differences between the groups were present, post hoc Tukey`s test for multiple comparisons was used to reveal differences between all pairs of groups. IL-6 concentration and total IL-6 quantity were not normally distributed. They were tested for global differences using a Kruskal–Wallis test followed by a post hoc Dunn’s multiple comparison test.

Correlation between AQP4 and IL-6 concentration in the preoperative CSF specimen was tested using Spearman`s correlation. Association of AQP4 and IL-6 with ventricular reduction was tested using the Chi square test. *P* values less than 0.05 were considered to be statistically significant (95 % confidence interval).

## Results

### Animals and clinical examination

Breed, age, sex, clinical signs and duration of clinical signs are summarised in Table 1 (Study group dogs) and Table 3 (Control dogs). Two dogs were excluded from the study group because there was no postoperative reduction of ventricular volume.

### CSF analysis

Results of CSF analyses are presented in Table 2 (study group dogs) and Table 3 (control dogs).

**Table 2:** Pre and post-operative concentrations for aquaporin 4 and interleukin 6 and CSF volumes for dogs with hydrocephalus

Dog number	Routine CSF examination	Pre-operative values AQP4 (ng/mL), total AQP4 (ng), IL-6 (IU/mL) total IL-6 (IU), CSF volume (mL)	Postoperative values AQP4 (ng/mL), total AQP4 (ng), IL-6 (IU/mL), total IL-6 (IU), CSF volume (mL)
1	*Protein* 252 mg/L *RBC* 0/μL *Cells* 3/μL	*AQP4* 12.78 *Total AQP4 quantity* 301.5 *IL-6* 63 *Total IL-6 quantity* 1486.8 *CSF volume* 23.6	*AQP4* 10.81 *Total AQP4 quantity* 28.51 *IL-6* 30 *Total IL-6 quantity* 84 *CSF volume* 2.8
2	*Protein* 276 mg/L *RBC* 280/μL *Cells* 1/μL	*AQP4* 10.77 *Total AQP4 quantity* 223.5 *IL-6* 87 *Total IL-6 quantity* 2260.8 *CSF volume* 26.4	*AQP4* 10.2 *Total AQP4 quantity* 27.99 *IL6* 36 *Total IL-6 quantity* 96.62 *CSF volume* 2.6
3	*Protein* 276 mg/L *RBC* 291/μL *Cells* 5/μL	*AQP4* 17.47 *Total AQP4 quantity* 391.33 *IL-6* 22 *Total IL-6 quantity* 492.8 *CSF volume* 22.4	*AQP4* 14.23 *Total AQP4 quantity* 150 *IL-6* 15 *Total IL-6 quantity* 61.1 *CSF volume* 10.6
4	*Protein* 240 mg/L *RBC* 0/μL *Cells* 1/μL	*AQP4* 15.29 *Total AQP4 quantity* 455.56 *IL-6* 28 *Total IL-6 quantity* 834.4 *CSF volume* 29.8	*AQP 4*9.71 *Total AQP4 quantity* 45.62 *IL-6* 13 *Total IL-6 quantity* 634.5 *CSF volume* 4.7
5	*Protein* 240 mg/L *RBC* 0/μL *Cells* 5/μL	*AQP4* 13.51 *Total AQP4 quantity* 278.3 *IL-6* 39 *Total IL-6 quantity* 803.4 *CSF volume* 20.6	*AQP4* 5.3 *Total AQP4 quantity* 34.45 *IL-6* 21 *Total IL-6 quantity* 136.5 *CSF volume* 6.5
6	*Protein* 208 mg/L *RBC* 0/μl *Cells* 1/μl	*AQP4* 10.33 *Total AQP4 quantity* 133.3 *IL-6* 51 *Total IL-6 quantity* 658.41 *CSF volume* 12.91	*AQP4* 9.9 *Total AQP4 quantity* 63.67 *IL-6* 32 *Total IL-6 quantity* 205.76 *CSF volume* 6.43
7	Blood contamination	*AQP4* 17.96 *Total AQP4 quantity* 467.67 *IL-6* 87 *Total IL-6 quantity* 2265.48 *CSF volume* 26.04	*AQP4* 8.93 *Total AQP4 quantity* 21.43 *IL-6* 37 *Total IL-6 quantity* 88.8 CSF volume 2.4
8	*Protein* 246 mg/L *RBC* 15/μL *Cells* 5/μL	*AQP4* 8.46 *Total AQP4 quantity* 211.54 *IL-6* 62 *Total IL-6quantity* 1078.8 *CSF volume* 17.4	*AQP4* 10.77 *Total AQP4 quantity* 3.95 *IL-6* 21 *Total IL-6 quantity* 13.4 *CSF volume* 1.64
9	Blood contamination	*AQP4* 12.16 *Total AQP4 quantity* 472.45 *IL-6* 65 *Total IL-6 quantity* 1709.5 *CSF volume* 26.3	*AQP4* 2.41 *Total AQP4 quantity* 58.03 *IL-6* 40 *Total IL-6 quantity* 260 *CSF volume* 6.5
10	*Protein* 300.9 mg/L *RBC* 18/μL *Cells* 7/μL	*AQP4* 19.19 *Total AQP4 quantity* 675.31 *IL-6* 66 *Total IL-6 quantity* 2323.2 *CSF volume* 35.2	*AQP4* 9.67 *Total AQP4 quantity* 190.95 *IL-6* 69 *Total IL-6 quantity* 1362.75 *CSF volume*:19.7
11	*Protein* 374 mg/L *RBC* 1/μL *Cells* 1/μL	*AQP4* 14.63 *Total AQP4 quantity* 168.85 *IL-6* 54 *Total IL-6 quantity* 623.16 *CSF volume* 11.54	*AQP4* 16.2 *Total AQP4 quantity* 58.31 *IL-6* 45 *Total IL-6 quantity* 162 *CSF volume* 3.6
12	*Protein* 204 mg/L *RBC* 0/μL *Cells* 4/μL	*AQP4* 7.1 *Total AQP4 quantity* 130.14 *IL-6* 89 *Total IL-6 quantity* 1631 *CSF volume* 18.33	*AQP4* 4.5 *Total AQP4 quantity* 42.93 *IL-6* 46 *Total IL-6 quantity* 438.84 *CSF volume* 9.54

Pre-, and post-operative determination of the ventricular volume (CSF volume), aquaporin-4 (AQP4) and interleukin-6 (IL-6) of the study group of 12 dogs with hydrocephalus. AQP4 and IL-6 have been multiplied by the total CSF volume, to give the total quantity in the CSF. The underlined entries (dogs 8 and 11) are dogs in which no decrease of AQP4 and IL-6 concentration was found after surgery although the total quantities were decreased (*CSF* cerebrospinal fluid, *RBC* red blood cell count)

**Table 3:** Epidemiological data and CSF analysis in the control group

Breed, age, gender, body weight	Routine CSF examination	CSF volume (mL)	AQP4 concentrations and total quantity	IL-6 concentrations and total quantity
Pug, 4 years male neutered, 4.6 kg	*Protein* 262 mg/L *RBC* 0/μL *Cells* 3/μL	2.4	AQP4: 11.25 *Total AQP4 quantity* 27	IL-6: 25 IU *Total IL-6 quantity* 60
Dachshund, 9 years, male, 6 kg	*Protein* 256 mg/L *RBC* 0/μL *Cells* 1/μL	3.2	AQP4: 9.67 *Total AQP4 quantity* 30.94	IL-6: 24 IU *Total IL-6 quantity* 76.8
Austrian hound, 3 years male, 26 kg	*Protein* 220 mg/L *RBC* 0/μL *Cells* 3/μL	3.14	AQP4: 9.0 *Total AQP4 quantity* 28.26	IL-6: 27 IU *Total IL-6 quantity* 85.87
German Shepherd dog, male, 5 years, 22 kg	*Protein* 302 mg/L *RBC* 0/μL *Cells* 2/μL	4.2	AQP4: 12.33 *Total AQP4 quantity* 51.78	IL-6: 19 IU *Total IL-6 quantity* 79.8
Beagle, male-neutered 2 years, 12 kg	*Protein* 286 mg/L *RBC* 0/μL *Cells* 1/μL	2.45	AQP4: 8.87 *Total AQP4 quantity* 21.74	IL-6: 42 IU *Total IL-6 quantity* 102.9
Doberman, male, 4 years, 36 kg	*Protein* 120 mg/L *RBC* 0/μL *Cells* 1/μL	3.04	AQP4: 6.57 *Total AQP4 quantity* 19.97	IL-6: 20 IU *Total IL-6 quantity* 60.8
French Bulldog female-neutered, 1,5 years, 8 kg	*Protein* 79 mg/L *RBC* 0/μL *Cells* 2/μL	3.4	AQP4: 9.1 *Total AQP4 quantity* 30.60	IL-6: 27 IU *Total IL-6 quantity* 91.8
Bernese mountain dog, female, 6 years, 34 kg	*Protein* 265 mg/L *RBC* 0/μL *Cells* 3/μL	4	AQP4: 11.97 *Total AQP4 quantity* 47.86	IL-6: 22 IU *Total IL-6 quantity* 88
Cavalier King Charles spaniel, female, 8 years, 6 kg	*Protein* 289 mg/L *RBC* 0/μL *Cells* 5/μL	3.2	AQP4: 11.52 *Total AQP4 quantity* 36.87	IL-6: 34 IU *Total IL-6 quantity* 108.8
Doberman, female- neutered, 7 years, 29 kg	*Protein* 156 mg/L *RBC* 0/μL *Cells* 1/μL	3.21	AQP4: 8.03 *Total AQP4 quantity* 25.79	IL-6: 55 IU *Total IL-6 quantity* 176.55

CSF volume, aquaporin-4 (AQP4) and interleukin-6 (IL-6) concentrations in the control group. Both values have been multiplied with the total CSF volume, expressed as AQP4* and IL-6* to obtain the total quantity in CSF (*CSF* cerebrospinal fluid, *RBC* red blood cell count)

#### AQP4

**Fig. 4:**
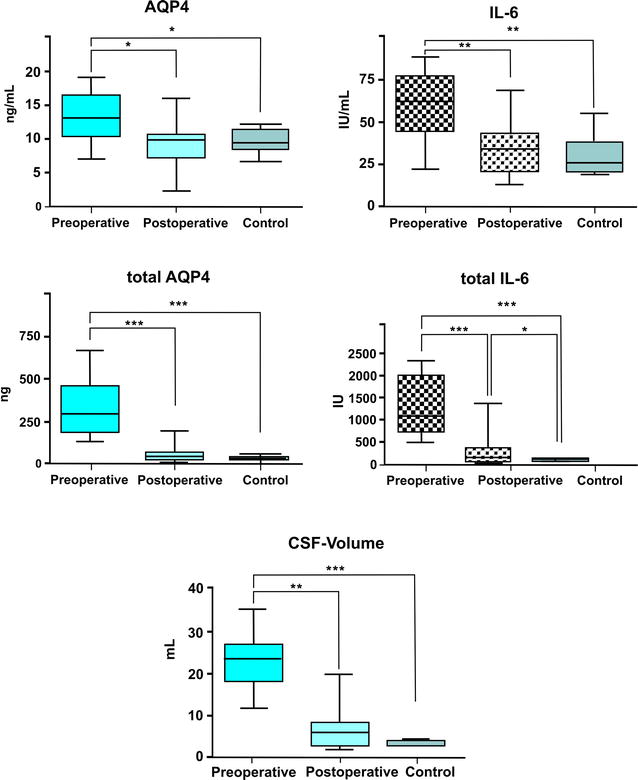
*Box* and *Whisker* diagrams demonstrating differences in the mean/median, 25/75 % percentile and minimum-maximum of the, aquaporin-4 (AQP4) and interleukin-6 (IL6) concentrations in the cerebrospinal fluid before and 3 months after surgery compared to controls (*top row*). The total quantity of both parameters AQP4 and IL6 (*middle row*) was calculated by multiplying by the ventricular volume (*bottom graph*). Significant differences are marked with *asterisks* (*P < 0.01; **P <0.001; ***P < 0.0001, n = 14, 14, 10 for preoperative, postoperative and control groups, respectively

Results of group comparisons are summarised in Fig. 4. The mean concentration of AQP4 was globally different between groups (*P* < 0.0127). Post hoc tests revealed a significant difference between the mean AQP4 concentrations before (11.32 ng/mL), and after surgery (9.3 ng/mL; *P* < 0.01), and before surgery compared to controls (9.5 ng/mL; *P* < 0.01). Postoperative AQP4 concentrations were not significantly different from controls (*P* > 0.05).

Total AQP4 quantities were globally different between groups (*P* < 0.0001). The mean total AQP4 quantity (325.8 ng) in dogs with internal hydrocephalus before surgery was significantly different from control dogs (32.12 ng, *P* < 0.0001). After shunting, mean total AQP4 quantity was significantly different from pre-operative values (60.49 ng, *P* < 0.0001), but not different from controls (*P* > 0.05). CSF volume was significantly reduced after surgery (*P* < 0.001). A reduction of ventricular volume was associated with AQP4 decrease (*P* < 0.001).

#### AQP1

AQP1 levels remained below the detection limit in all CSF samples analysed. Using an AQP1 ELISA kit and the same procedure as for AQP4, OD values were around zero.

#### IL-6

IL-6 concentrations (Fig. 4) were globally different between the groups (*P* < 0.0036). The median IL-6 concentrations before surgery (62 IU/mL) was significantly higher than postoperative values (34 IU/mL; *P* < 0.001) and higher when compared to controls (26 IU/mL; *P* < 0.001). Postoperative values were not different to controls (*P* > 0.05). Total IL-6 quantities were significantly different between groups (*P* < 0.0001). Total IL-6 quantities were significantly higher in dogs with internal hydrocephalus than in control dogs before surgery (1083 vs. 86.3 IU; *P* < 0.0001) and significantly decreased after shunting (149.3 IU; P < 0.0001), but were still significantly higher than in the control group (*P* < 0.01).

Concentrations of AQP4 and IL-6 measured in the preoperative CSF specimens were not correlated (*P* = 0.449).

## Discussion

We found increased AQP4 and IL-6 concentrations in the CSF of dogs with idiopathic communicating hydrocephalus. AQP4 and IL-6 concentrations decreased significantly after reduction of lateral ventricular volume using an indwelling ventriculo-peritoneal shunt system. Up-regulation of AQP4 channels has been documented in a variety of pathological processes in the brain that result in fluid overload, including internal hydrocephalus. In rats with inherited [19] and kaolin-induced hydrocephalus [15], an increase in AQP4 mRNA- and protein levels within the periventricular parenchyma was reported post-induction. It seems likely that the increase in AQP4 reflects the development of an alternative pathway for parenchymal CSF absorption [9, 15–17, 19, 20]. This notion is also supported by the observation that AQP4 null mice exhibit a more severe form of hydrocephalus with larger ventricular distension and pressure elevation after kaolin injection, than mice with unimpaired AQP4 expression [29]. Whereas AQP4 changes have been well described in experimentally-induced hydrocephalus, few publications have studied the association between AQPs and naturally-occurring hydrocephalus. This is important in that hydrocephalus is usually non-communicating in laboratory rodents, but communicating in the dogs reported in the present study. Hence, there are more similarities between human and canine communicating hydrocephalus, when compared to experimentally-induced hydrocephalus after intrathecal kaolin injection in rodents and the subsequent inflammatory reaction. It has been shown in dogs with induced hydrocephalus that after an initial rise, CSF pressure can return to normal levels as the ventricles enlarge [30].

In the classic model of CSF physiology in dogs the fluid is produced by the choroid plexus [31]. The primary sites of reabsorption are the arachnoid projections from the subarachnoid space into the sagittal dural venous sinus. There is evidence that extracellular fluid (ECF) from the brain parenchyma essentially contributes to CSF production and its bulk flow within the central nervous system [32]. Under normal conditions, arterial pulsation drives the ECF toward the veins and towards the ventricles in humans and dogs. It has been suggested that this physiological ECF flow is reduced in hydrocephalus, and the pericapillary space has been confirmed as playing a critical role in the reabsorption of ECF [9, 33]. The ependymal lining is frequently destroyed in hydrocephalus [34] and AQP4 can, therefore, leak more easily from the parenchyma to the CSF via the ECF [18].

It remains unclear however, whether the increased AQP4 levels in the CSF of hydrocephalic dogs and humans is an unspecific finding related simply to the destruction of cell membranes. In this scenario, on the one hand, an increase of AQP4 would be a pure epiphenomenon reflecting damage to the structural integrity of the ependyma [35] and periventricular white matter. This might be the more likely cause of the increase in AQP4 in the CSF. On the other hand, it has also been suggested that higher AQP4 levels could also be a reflection of increased production and turnover of the protein and its release into the interstitial space and ventricular system. Such shedding of membrane proteins has been documented for renal AQPs. Wen et al. [36] showed that urinary excretion of AQP2 occurs under physiological conditions as part of its increased integration into the apical membrane of the collecting duct after vasopressin stimulation. During this process AQP2 is excreted into the urine in a proportion specific to the quantity of membrane-bound AQP [37–40].

It has been shown that distension of the ventricles and compression of periventricular white matter capillaries are accompanied by pro-inflammatory cytokine activation. Damage to the periventricular white matter is the main impact on brain integrity in hydrocephalus cases [34, 41]. IL-6 has been associated with periventricular white matter injury in newborn babies with peripartal hypoxia [22] and increases in IL-6 and IL-8 were more pronounced in the infants with a severe clinical course [42]. A correlation between IL-6 levels and white matter integrity has been documented in elderly humans [43, 44]. We therefore consider that changes in IL-6 concentrations in the CSF act as a surrogate marker for white matter injury in the present study. Here, it has become evident that AQP4 concentration was not correlated with IL-6 concentration in CSF taken preoperatively. This observation might favour the notion that CSF AQP4 level does not merely reflect white matter damage, however, the exact circumstances between pressure rise and AQP4 changes in the CSF remain to be further analysed in future studies.

Our study also showed that adjusting the concentrations of AQP4 and IL-6 to the total CSF volume was not necessary to detect significant differences in AQP4 and IL-6 concentrations. Following the rationale that CSF is constantly produced and the amount of the solute may depend on the amount of the total solvent (CSF), we considered it might be necessary to calculate AQP4 and IL-6 total quantities by multiplying by the ventricular volume. Although preoperative volumes in some dogs exceeded three times the volume of other hydrocephalic dogs, the differences in ventricular dimensions did not obscure differences in CSF AQP4 concentrations. In fact, the concentrations were comparable to those measured in children with hydrocephalus (13.3 in dogs vs. 11.32.ng/mL in children), as well as those in normal dogs to the values in unaffected children (9.5 in dogs vs. 8.61 ng/mL in children) [18].

The main limitation of this study is therefore the heterogeneity of the examined dogs. Different dog breeds were examined without knowledge of the underlying cause of the CSF accumulation or of intraventricular pressure, with different degrees of ventricular dilatation and different durations of clinical signs. Dogs with hydrocephalus were younger than control dogs. Furthermore, CSF from the control group was taken after euthanasia and also not from the intraventricular site, which might also have a potential influence on measured AQP4 and IL-6 levels.

While we were able to measure AQP4 in the CSF of normal and hydrocephalic dogs, AQP1 levels were below the detection limit in all samples. Interestingly, analysis of AQP1 expression after the induction of hydrocephalus in rodents has revealed conflicting results. In mice, it has been shown that AQP1 is down-regulated after induction of hydrocephalus, rather suggesting a compensatory response to hydrocephalus [41], however, another study showed unchanged AQP1 expression in a rat model of hydrocephalus [15]. Being confined to the choroid plexus, AQP1 expression is much lower than AQP4 expression in the rodent brain and CSF. This is also reflected in the fact that values remained below the detection limit in our current analyses for canine CSF AQP1.

## Conclusions

An increase in ventricular dimension is accompanied by increases in AQP4 and IL-6 concentrations in the CSF of dogs with idiopathic communicating hydrocephalus. All were greatly reduced or normalised after shunt treatment. Therefore AQP4 and IL-6 in CSF can be indicators of ventriculomegaly, of cellular damage, and of improvement after the treatment of hydrocephalus. Based on the results of this study, a brain tissue-based determination of AQP4 at the mRNA and protein level might be rewarding to analyse the potential role of AQP4 in the compensation of extracellular fluid overload in dogs with communicating hydrocephalus.
